# New hope for Parkinson's disease treatment: Targeting gut microbiota

**DOI:** 10.1111/cns.13916

**Published:** 2022-07-13

**Authors:** Hong‐Xia Fan, Shuo Sheng, Feng Zhang

**Affiliations:** ^1^ Laboratory Animal Center and Key Laboratory of Basic Pharmacology of Ministry of Education and Joint International Research Laboratory of Ethnomedicine of Ministry of Education and Key Laboratory of Basic Pharmacology of Guizhou Province Zunyi Medical University Zunyi Guizhou China

**Keywords:** *Desulfovibrio*, *Enterococcus faecalis*, gut microbiota, Parkinson's disease

## Abstract

There might be more than 10 million confirmed cases of Parkinson's disease (PD) worldwide by 2040. However, the pathogenesis of PD is still unclear. Host health is closely related to gut microbiota, which are affected by factors such as age, diet, and exercise. Recent studies have found that gut microbiota may play key roles in the progression of a wide range of diseases, including PD. Changes in the abundance of gut bacteria, such as *Helicobacter pylori*, *Enterococcus faecalis*, and *Desulfovibrio*, might be involved in PD pathogenesis or interfere with PD therapy. Gut microbiota and the distal brain achieve action on each other through a gut‐brain axis composed of the nervous system, endocrine system, and immune system. Here, this review focused on the current understanding of the connection between Parkinson's disease and gut microbiota, to provide potential therapeutic targets for PD.

## Graphical abstract

We reviewed the relationship between Parkinson's disease and gut bacteria, especially *Helicobacter pylori*, *Enterococcus faecalis*, and *Desulfovibrio*. At the same time, three kinds of dialogue mechanisms between brain and gut are also discussed. Finally, three potential interventions for gut microbiota are discussed.

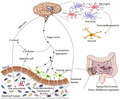

## 1 INTRODUCTION

Parkinson's disease (PD) is one of the common neurodegenerative diseases, with more than 10 million confirmed cases of PD estimated worldwide by 2040.^1^ Tremor, rigidity, and bradykinesia are typical clinical manifestations of PD.^2^ Non‐motor symptoms include gastrointestinal dysfunction, such as constipation, sleep behavior disorder, depression, and cognitive impairment.^3^ Importantly, non‐motor symptoms and motor symptoms might influence each other to aggravate PD severity,^4^ thereby exacerbating the deterioration of quality of life and mentality in patients with PD.

The pathogenesis of PD is complex, which is mainly related to complex environmental factors. A large number of epidemiological studies indicated that the prevalence of PD was higher in people over 50 years old, and the incidence increased with age.^5^ The prevalence of PD was higher in men than that in women at the same age.^6^ However, there were significant differences in PD prevalence among different regions and races in different studies.^7^, ^8^, ^9^ In addition, current evidence demonstrated that genetic factors were only associated with a small number of PD cases.^10^ Moreover, PD was more affected by environmental factors, such as pesticide exposure and dairy intake. On the other hand, traumatic brain injury and β2‐adrenergic receptor antagonists were recognized to be risk factors that increased the probability of PD, while nicotine in tobacco, caffeine in coffee, tea, and anti‐inflammatory medication (non‐steroidal anti‐inflammatory drugs, statins) intake had a negative correlation with the incidence of PD.^11^, ^12^, ^13^

When PD patients showed obvious clinical symptoms, the dopaminergic (DA) neurons containing neuromelanin (NM) in the substantia nigra (SN) of midbrain were damaged.^14^ The pathological features were the accumulation of iron in SN, loss of DA neurons in the ventrolateral and caudal layers, and the increased levels of extracellular NM.^15^ Furthermore, NM interacted with excess Fe^3+^ ^16^, ^17^ and then combined with α‐synuclein in the cytoplasm^18^ to damage DA neurons by destroying Ca^2+^ ^19^ homeostasis or activating microglia.^20^, ^21^

Currently, several imaging techniques are used to evaluate PD. For example, advanced magnetic resonance imaging (diffusion, iron‐sensitive, and neuromelanin‐sensitive sequences) can detect the changes in the brain microstructure of PD patients.^15^, ^22^ PET imaging with 18F‐DOPA to quantitatively estimate nigrostriatal DA neurodegeneration.^23^ SPECT imaging using radiolabeled tracers that bind dopamine transporter (DAT) ligands ([^123^I] FP‐CIT, [^123^I] β‐CIT, [^99m^Tc]–TRODAT‐1) to determine DAT activity and help assess the integrity of the DA substantia nigra pathway.^24^ Detection of early changes in the SN region by the above means could be used as a supplement method to the clinical diagnosis of PD.^25^ In addition, PD can be distinguished from atypical PD by cerebral magnetic resonance imaging.^26^ Symptoms of dementia may interfere with the diagnosis of PD, and DAT‐SPECT is used to discriminate between Alzheimer's disease and Lewy body dementia.^25^

In addition, the hyperthyroidism symptoms caused by L‐thyroxin partially overlap with PD symptoms, which may interfere with the diagnosis of PD. The loss of dopaminergic neurons in the substantia nigra in PD was irreversible. Tremors and other motor disturbances occurred due to the damaged substantia nigra‐striatum dopamine projection pathway.^27^ Administration of levodopa can only attenuate the symptoms of PD but not delay the development of PD. L‐thyroxin was used as a thyroid replacement therapy for hypothyroidism. However, excessive use of L‐thyroxin could lead to hyperthyroidism,^28^ which increased the metabolic rate and caused symptoms, including sweating, anxiety, tachycardia, and tremors.^29^, ^30^ This can be blocked by discontinuing the use of L‐thyroxin or using β–blockers, such as propranolol, atenolol, and metoprolol.^31^ Various animal studies indicated that L‐thyroxin‐induced hyperthyroidism resulted in the increased levels of malondialdehyde and glutathione in rat brain tissue, which further activated the oxidant and anti‐oxidant system.^32^, ^33^ However, excessive lipid peroxidation was thought to be a hallmark of most neurodegenerative diseases, such as PD.^34^ In addition, the incidence of hypothyroidism and hyperthyroidism in L‐thyroxin‐treated PD patients was exhibited to be higher than that in the control group, and hyperthyroidism was also closely associated with higher PD risk.^35^, ^36^

Rapid advances in high‐throughput technology over the past 20 years have led to a great understanding of the number, composition, and function of intestinal bacteria. Homeostasis of intestinal bacteria is crucial for maintaining the health of the host. However, the gut flora is susceptible to changes in host age, diet, and exercise.^37^, ^38^, ^39^ At present, a large number of studies have suggested that intestinal bacteria may be involved in the pathogenesis of neurodegenerative diseases, such as Alzheimer's disease, amyotrophic lateral sclerosis, and PD.^40^, ^41^, ^42^ Furthermore, changed intestinal microecology has also been observed in patients with diseases associated with PD, such as diabetes and colitis.^43^, ^44^ In‐depth understanding of the association between intestinal bacteria and PD may lead us to find potential therapeutic targets for PD in the gut, which is conducive to the development of treatments for PD.

## 2 INTERSECTION OF GUT MICROBIOTA AND PARKINSON'S DISEASE

There are currently approximately 1000 ubiquitous species in the human gut, reaching a total number of 3.8 × 10^13^.^45^, ^46^ Mammals have a complex symbiotic relationship with their gut microbiota. Host‐secreted microRNAs (miRNAs) are non‐coding RNA molecules that participate in transcription. Recent studies have found that miRNAs could conduct intercellular translocation through gap‐cell contact or exosome release,^47^ and then enter bacterial cells and regulate growth behavior and bacterial gene transcripts.^48^ For example, miRNA‐146A, (HSA)‐miRNA‐515‐5p, and HSA‐miRNA‐1226‐5p were verified to correlate with the abundance of *Listeria*, *Fusobacterium nucleatum*, and *Escherichia coli* (*E. coli*), respectively.^49^, ^50^ In addition, Firmicutes and Proteobacteria were presumed to have the highest potential target site of miRNA. Meanwhile, several miRNA‐targeted proteins (hsa‐miR‐3180‐5p, hsa‐miR‐132‐5p, and hsa‐miR‐3180‐5p) were involved in lipopolysaccharide biosynthesis and biofilm formation pathways of various pathogenic bacteria in the pathogenesis of PD.^47^ In turn, bacterial infections, such as *Mycobacterium tuberculosis*, *Helicobacter pylori* (*H. pylori*), and *Salmonella enterica*, also regulated host miRNA expressions and thereby host immune function.^48^ These results suggest that there might be crosstalk between host and symbiotic bacteria.

Additionally, another evidence confirmed the significant differences of gut microbiota between PD and healthy people. In detail, the intestinal microbiome richness (Observed and Chao 1) in PD patients was decreased compared with healthy controls by sequencing the V4 and V5 regions of bacterial 16S rRNA genes. The relative abundance of *Clostridiales* family XI and the genus *Peptoniphilus* was high in PD patients, while genera *Faecalibacterium* and *Fusicatenibacter* were decreased.^51^ Interestingly, the intestinal microbiota of PD patients treated with drugs also differed from that in healthy controls. An apparent increase in the relative abundance of *Peptoniphilus* and *Finegoldia* genera in PD patients treated with levodopa was shown, while the relative abundance of *Bifidobacteriaceae*, *the genera Peptoniphilus*, *Anaerococcus*, *the Eubacterium brachy group*, *Sellimonas*, *Bifidobacterium*, and *Enterococcus* was increased in PD patients treated with entacapone. Moreover, the relative abundance of Enterococcaceae and Clostridiales family XI was increased in PD patients treated with levodopa or Entacapone, while Faecalibacterium and the Ruminococcus gauvreauii were decreased.^51^

It should also be noted that human gut contains not only bacteria but also incompletely identified eukaryotes. Recently, the richness of eukaryotes (Observed, Shannon, and Simpson) was found to be reduced in PD patients by sequencing the V6 and V7 regions of the eukaryotes' 18S rRNA genes. Furtherly, the lower relative abundance of sequences affiliated with Aspergillus/Penicillium, Charophyta/Linum, unidentified Opisthokonta, and three genera of minor abundant zooflagellates in PD patients was discerned. At the same time, the abundance of Geotrichum was increased in PD patients treated with levodopa or Entacapone compared with healthy controls.^52^

Currently, the intestinal inflammation was considered to be an important contributor in the development of PD. Patients with inflammatory bowel disease (IBD) were at a higher risk of PD occurrence.^53^ Furthermore, PD patients had higher levels of calprotectin, a fecal marker of intestinal inflammation, as well as α1‐antitrypsin and zonulin, two fecal markers of intestinal permeability.^54^ Intestinal flora appeared to be involved in the formation of a relevant pro‐inflammatory intestinal environment. The relative abundance of *Peptoniphilus* and *Finegoldia*, which could cause a variety of microbial infections and inflammation, increased in calprotectin‐positive PD patients. However, *Faecalibacterium* and *Fusicatenibacter* were found to have anti‐inflammatory effects and the relatively low abundance of these 2 bacteria in calprotectin‐positive PD patients.^51^

Gut microbiota are involved in the metabolism and immune regulation of the host. Based on the critical role of metabolism and immunity in the development of PD, we summarized three intersections of PD pathogenesis and intestinal microbiota changes.

The growth of age is a natural change, which is accompanied by the development, maturation, and aging of the body's organs. Similarly, there is such a dynamic change process in gut microbiota. Studies have shown significant differences in the composition of gut microbiota among infants, adults, and the elderly.^55^, ^56^ The microbial community of healthy adults is heterogeneous, Firmicutes dominate, and the intestine has a stable microecology, while aging can disrupt this microecological balance and cause the reproduction of a large number of pathogenic bacteria. A recent study showed that PD patients had lower Simpson index and Equitability Index, suggesting dysregulation of gut microbiota in PD patients.^57^ Observed a decreasing trend in the abundance of Firmicutes, while the abundance of Bacteroidetes and Proteobacteria increased in subjects over 70 years of age susceptible to PD. Many common pathogens have been found in Proteobacteria and Bacteroidetes, such as *E. coli*, *Proteus mirabilis*, and *Bacteroides fragilis*, which could release lipopolysaccharide (LPS) as neurotoxins.^58^, ^59^ These neurotoxins may be rapidly or chronically released during aging and induced systemic inflammatory pathologies and Parkinson's disease.^60^, ^61^

The gut microbiota plays an active role in promoting the absorption and utilization of food. Due to the microbiota could induce glycosylation of small intestinal tissue factors and increase surface localization, the microbiota‐induced extravascular tissue factor protease‐activated receptor 1 signaling loop was considered to be a novel pathway that influenced vascular remodeling in the small intestine.^62^ Thus, the increased villi vascularization in the small intestine would promote the oxygen cooperation of villi and change villi morphology, which might ultimately affect the nutrient absorption of food by the host.^63^ In turn, the abundance of the gut microbiome fluctuates in response to food. Interestingly, different kinds of food could selectively affect the abundance, as well as metabolism of specific bacteria. For example, soybean intake was negatively associated with *Prevotella* and white bread intake increased *Bifidobacterial* abundance.^64^, ^65^ Further, dietary fiber and whole‐grain intake increase the abundance of related gut bacteria that produce short‐chain fatty acids (SCFAs).^66^, ^67^ SCFAs is an important energy source for colonic epithelial cells and can enhance the integrity of the epithelial barrier.^68^ Meanwhile, SCFAs are signal molecules that connect microbe and host, and their receptors are highly expressed in immune cells. In addition, some SCFAs can cross the blood–brain barrier, regulate the permeability of the blood–brain barrier, and restore the number of damaged microglia as well as their function and morphology.^69^

Studies have shown that dietary fat was closely associated with the increased intestinal permeability. Tight junction protein and mucin constitute the apical junctional complex, which connects intestinal epithelial cells and forms an important barrier between host and environment. The intestinal barrier selectively controls the infiltration of nutrients and prevents the infiltration of pathogenic substances.^70^ High‐fat diet (HFD) not only reduces the expression of intestinal tight junction protein, but also stimulates the intracellular protein kinase C signaling pathway to increase intestinal wall cell permeability.^71^, ^72^ In addition, dietary fat elicited the synthesis of hydrophobic bile acids (deoxycholic acid and deoxycholic acid), both of which stimulate EGFR‐mediated signaling and enhance ROS‐induced oxidative stress to further promote intestinal permeability.^73^, ^74^, ^75^ At the same time, HFD was associated with a decrease in intestinal bacterial diversity, and HFD increased the relative abundance of Enterobacteriaceae and decreased the relative abundance of Bacteroidetes.^76^, ^77^ Although both Enterobacteriaceae and Bacteroidetes were gram‐negative bacteria, the lipid A structure of Enterobacteriaceae bound to members of the TLR4‐activated pathway better than the lipid A structure of Bacteroidetes.^77^, ^78^ Therefore, HFD‐mediated increased intestinal permeability could promote passive translocation of LPS or LPS‐containing gram‐negative bacteria, resulting in more LPS translocation into the blood circulation system to trigger systemic inflammation.^70^

Exercise can produce changes in gut microbial composition and play an active role in energy homeostasis and microbiota regulation.^79^, ^80^ A recent clinical studies suggest that high‐intensity aerobic exercise improved PD symptoms and bimanual dexterity.^81^ This may be because exercise increases the reproduction of SCFAs producing bacteria. Aerobic exercise has been found to increase the concentration of SCFAs in feces, and these short‐chain fatty acid levels decrease again after cessation of exercise.^82^ Therefore, healthy microbiome composition and metabolism require appropriate and sustained exercise to maintain.

The above results suggest that a new protective intervention for PD can be developed by increasing the colonization of specific intestinal bacteria through a fiber‐rich diet and regular exercise.

## 3 GUT BACTERIA ASSOCIATED WITH PARKINSON'S DISEASE

PD is characterized by degeneration of dopaminergic neurons in the SN, formation of intracytoplasmic eosinophilic inclusions (Lewy bodies, mainly composed of abnormal deposition of α‐synuclein), destruction of the nigrostriatal pathway, and decreased dopamine content in the striatum.^83^ Studies have found that α‐synuclein structures exist in the colon of patients with early PD and also α‐synuclein is deposited as early as before the clinical diagnosis of PD patients,^84^ suggesting that the accumulation of intestinal α‐synuclein preceded the onset of PD, and PD may begin in the intestine.^85^ At the same time, studies have found that gastrointestinal dysfunction in PD patients occurs many years earlier than PD,^86^ suggesting a close link between intestinal diseases and PD. For example, a higher association between IBD and the risk of PD was presented.^87^, ^88^ Thus, the alterations of microbiota in PD^89^ might exist in the bidirectional relationship of bacteria/PD, bacteria/gastrointestinal symptoms, and gastrointestinal symptoms/PD.

### 3.1 Helicobacter pylori

*Helicobacter pylori* is located in the intestinal epithelium, one of the common bacterial infections. Studies have found that PD patients had a high prevalence of *H. pylori* infection.^90^, ^91^, ^92^, ^93^ A study showed a close association between *H. pylori* positivity and worse PD exercise severity.^94^ In 102 PD patients, the degree of motor symptoms was heavier in *H. pylori*‐positive group than that in *H. pylori*‐negative group by assessing the clinical symptoms of the patients. In addition, the mean UPDRS‐III score was reduced after *H. pylori* eradication therapy.^95^

*Helicobacter pylori* infection was highly associated with the duodenum, which is the main site of levodopa absorption.^96^ One meta‐analysis found that PD patients with *H. pylori* infection had higher levodopa equivalent daily dose than those of patients without *H. pylori* infection.^97^ Moreover, a prospective study exhibited that *H. pylori* eradication improved levodopa onset time, ‘ON’ duration, motor severity, and quality of life parameters.^98^ *H. pylori* might utilize levodopa to promote its own growth, thus reducing the amount of oral levodopa used to treat PD‐related pathologies.^99^ However, a recent randomized placebo‐controlled trial displayed that eradication of *H. pylori* did not improve the clinical outcome of PD.^100^ The conflicting conclusions suggest that further studies are needed to demonstrate that *H. pylori* eradication has important therapeutic implications for PD patients.

### 3.2 Enterococcus faecalis

Levodopa is the drug used for dopamine replacement therapy in PD treatment regimens. Since levodopa is metabolized peripherally, it is combined with decarboxylase inhibitors to improve its utilization. Despite this, levodopa/decarboxylase inhibitors are ineffective in some PD patients, and their efficacy decreases over time of treatment, requiring frequent drug dose changes.^101^ It was reported that fecal tyrosine decarboxylase (TyrDC) abundance was positively correlated with levodopa dose and levodopa levels in plasma were negatively correlated with the abundance of bacterial TyrDC genes in the jejunum of PD patients.^102^ On the one hand, TyrDC was identified mainly in *Enterococcus faecalis*, which decarboxylated L‐tyrosine to tyramine, while tyrosine differed from levodopa by only one additional hydroxyl group.^103^ On the other hand, genetic and biochemical experiments showed that TyrDC decarboxylated both levodopa and its preferred substrate tyrosine, which accounted for the variable efficacy of levodopa.

Recent studies confirmed that *Enterococcus faecalis* could decarboxylate both levodopa and tyrosine. *Enterococcus faecalis* and carbidopa did not affect each other's decarboxylated effects on levodopa.^101^ Therefore, in PD patients with large numbers of *Enterococcus faecalis*, the serum peak concentration of levodopa will be reduced. Thus, the abundance of *Enterococcus faecalis* and its coding TyrDC can predict the interindividual differences in levodopa metabolism in complex human gut microbiome samples. Therefore, *Enterococcus faecalis* is also one of the factors affecting the bioavailability of Levodopa, and inhibition of *Enterococcus faecalis* may be one of the means to improve the bioavailability of Levodopa, which has the potential to treat PD.

### 3.3 Desulfovibrio

Sulfate‐reducing bacteria is a kind of anaerobic bacteria that obtains energy through dissimilated sulfate reduction, thus producing a large amount of sulfide.^104^ Human isolates of sulfate‐reducing bacteria consist almost exclusively of *Desulfovibrio* (*DSV*) species.^105^, ^106^ *DSV* is associated with IBD and bacteremia infections.^104^, ^107^ Results from a case–control study showed that the relative abundance of Desulfovibrionaceae bacteria was increased in the microbiota of PD patients.^108^

*DSV* has two properties that are closely related to PD pathogenesis. On the one hand, *DSV* could produce hydrogen sulfide (H_2_S) by dissimilatory sulfate reduction using sulfate as electron acceptor for respiration.^109^ H_2_S has been involved in multiple signaling mechanisms under normal physiological conditions.^110^ However, high dose of H_2_S gas exposure resulted in severe central nervous system (CNS) dysfunction and even death.^111^, ^112^ At the same time, H_2_S released mitochondrial cytochrome c into the cytoplasm, and the peroxidase activity of cytochrome c contributed to the formation of α‐synuclein free radicals, which triggers α‐synuclein oligomerization.^113^, ^114^ In addition, H_2_S could interfere with iron metabolism by increasing iron content in the cytoplasm.^115^, ^116^ Iron homeostasis is essential in CNS. Additional evidence indicated that iron could induce α‐synuclein fibrosis.^117^, ^118^, ^119^ On the other hand, *DSV* is able to reduce ferric iron to ferrous iron by a periplasmic [FeFe]‐hydrogenase present in almost all *DSV*, thus producing magnetite (Fe_3_O_4_).^120^, ^121^ According to relevant studies, uncoated magnetite nanoparticles accelerated α‐synuclein aggregation.^122^

Recent studies suggested a close association between *DSV* bacteria and PD. The number of *DSV* bacteria in stool samples from PD patients correlated with the severity of PD and higher numbers of *DSV* were found in PD samples and all stool samples from PD patients were positive for the *DSV*‐specific [FeFe] ‐hydrogenase gene. In addition, the statistical results also showed that the amount of *DSV* was higher in PD patients with constipation.^123^ Constipation is one of the prodromal symptoms of PD, so the change of DSV content can be used as one of the indicators to predict the progression of PD.

### 3.4 Other bacteria

In addition to the above specific bacteria, the role of other bacteria on the pathogenesis of PD has also been verified. For example, *Nocardia asteroides* were found to induce parkinsonism‐like pathological symptoms in experimental mice as early as 1991.^124^ *Proteus mirabilis* isolated from PD mice selectively damaged dopaminergic neurons in the substantia nigra and striatum, and stimulated the accumulation of α‐synuclein in the brain and colon of PD model mice.^58^ *Citrobacter rodentium*, a murine enteric pathogen that mimics human pathogenic *E. coli* infection, was found to reduce spontaneous motility and motility defects in open field chambers after Pink1^−/−^ mice were exposed to *Citrobacterum* three times for more than 4 months.^125^ *Porphyromonas gingivalis* was able to damage dopaminergic neurons and increase microglia activation in the SN of LRRK2 R1441G mutant mice with the mRNA expressions of tumor necrosis factor and interleukin‐1β increased and the protein levels of α‐synuclein and occludens‐1 decreased in the colon.^126^ *Cyanobacteria* are associated with the pathogenesis of PD since they produce β‐N‐methylamino‐L‐alanine.^127^ β‐N‐methylamino‐L‐alanine is a neurotoxin that lowers the levels of glutathione, a major antioxidant, by activating metabotropic glutamate receptors.^128^ These studies show the contribution of different pathogenic bacteria to the progression of PD (Table 1).

**TABLE 1:** Effects of pathogens on the pathogenesis of Parkinson's disease

Pathogens	Effects	Mechanism
*Helicobacter pylori*	Parkinsonism: differential age‐trend in *Helicobacter pylori* antibody^90^ *Helicobacter pylori* eradication and levodopa absorption in patients with PD and motor fluctuations^91^ *Helicobacter pylori* infection and motor fluctuations in patients with Parkinson's disease^92^ *Helicobacter pylori* infection was related to a poor response to levodopa in patients with Parkinson's disease^97^	*Helicobacter pylori* interferes with levodopa absorption.
*Enterococcus faecalis*	Gut bacterial tyrosine decarboxylases restrict levels of levodopa in the treatment of Parkinson's disease^102^ Discovery and inhibition of an interspecies gut bacterial pathway for levodopa metabolism^101^	*Enterococcus faecalis* decarboxylates levodopa to dopamine in the gut.
*Desulfovibrio*	Gut microbiota in patients with Parkinson's disease in southern China^108^ *Desulfovibrio* bacteria were associated with Parkinson's disease^123^	*Desulfovibrio* Bacteria produce extracellular magnetite and H_2_S, both of which induce oligomerization and aggregation of α‐synuclein.
*Nocardia asteroides*	Levodopa‐responsive movement disorder caused by *Nocardia asteroides* localized in mouse brains^124^	*Nocardia asteroides* induce the formation of Lewy body‐like hyaline inclusions.
*Proteus mirabilis*	Oral administration of *Proteus mirabilis* damaged dopaminergic neurons and motor functions in mice^58^	*Proteus mirabilis* induces inflammation and stimulates α‐synuclein aggregation in the brain and colon.
*Citrobacter rodentium*	Characterization of the intestinal microbiota during *Citrobacter rodentium* infection in a mouse model of infection‐triggered Parkinson's disease^125^	*Citrobacter rodentium* induces the presentation of mitochondrial antigens on MHC I of Pink1^−/−^ mice and the subsequent formation of anti‐mitochondrial CD8+ T cells into the central nervous system.
*Porphyromonas gingivalis*	Oral *P. gingivalis* impaired gut permeability and mediated immune responses associated with neurodegeneration in LRRK2 R1441G mice^126^	*Porphyromonas gingivalis* induced high expression of inflammatory factors and increased α‐synuclein in R1441G mice.
*Cyanobacteria*	Microbial β‐N‐methylamino‐L‐alanine (BMAA) and the pathway for Parkinson's Disease^127^	BMAA produced by *cyanobacteria* may lead to protein misfolding and mitochondrial dysfunction.

In addition to the changes and effects of single bacteria, there have been many case–control studies focused on the changes of overall intestinal bacteria in PD patients recently, showing that the abundance of Prevotellaceae, Faecalibacterium, and Lachnospiraceae was lower in PD patients than in healthy controls. Meanwhile, a higher abundance level of Bifidobacteriaceae, Ruminococcaceae, Verrucomicrobiaceae, and Christensenellaceae was also found in patients with PD.^129^ A large number of studies exhibiting these altered gut bacteria have also been found to correlate with the severity of PD development. The relative abundance of Enterobacteriaceae was positively correlated with the severity of postural instability and gait difficulties and the number of bacteria in Enterobacteriaceae was much more in PD patients with predominant postural gait disorders than those in PD patients with predominant tremor.^89^ Additionally, correlation analysis confirmed that the low counts of *Bifidobacteria* and *Bacteroides fragilis* were closely related to the deterioration of the *UPDRS*‐I scores within 2 years.^130^ These findings suggest that gut microbiome in PD patients distinguished from healthy individuals, and these differences are highly correlated with the motor phenotype of PD.

## 4 COMMUNICATION BRIDGE OF GUT‐BRAIN AXIS

### 4.1 Nervous system

The classical Braak hypothesis thinks that the vagus is the conduit through which gut‐deposited α‐synuclein enters CNS.^131^ This hypothesis was confirmed by vagotomy. Intestinal bacteria participate in the communication of the gut‐brain axis as an indispensable part of the gastrointestinal tract. This bidirectional communication involves multiple nervous systems and hypothalamic pituitary adrenal (HPA) axis. The large and complex enteric nervous system (ENS) is able to coordinate gastrointestinal behavior independent on CNS.^132^ Gut has direct neural connections to brain through the vagus, and bacteria could stimulate afferent neurons in ENS.^133^ The connections between ENS and CNS are made by vagal and pelvic nerves and sympathetic pathways.^134^

The production of curli fimbriae, a common bacterial ingredient, is related to *E. coli*.^135^ Various animal studies have demonstrated that exogenous α‐synuclein fibrils, whether derived from PD patients or produced in *E. coli*, are able to form Lewy body‐like inclusions that spread from the gastrointestinal tract through the vagus to the rat brain.^136^

A large epidemiological study was conducted in Denmark, which selected 5339 patients who underwent vagotomy and 5870 patients who underwent highly selective vagotomy, and found that patients who underwent truncal had a reduced risk of PD compared with superselective vagotomy through 20 years of follow‐up. In addition, gut microbes could also exert beneficial effects on the brain by ascending through the vagus. One evidence indicated that vagotomy in mice blocked the effects of lactic acid bacteria, such as *Lactobacillus rhamnosus*, against anxiety‐related behaviors in mice.^137^ Similarly, the anxiolytic effects of *Bifidobacteria* on mice also disappeared after vagotomy.^138^

### 4.2 Immune system

In the communication of the brain‐gut axis, it is usually the nervous system, immune system, and endocrine system that cooperate to build a bridge between brain and gut communication. Innate immunity senses the presence of microorganisms through various pattern recognition receptors (PRRs) that specifically recognize evolutionarily conserved molecular structures, called pathogen‐associated molecular patterns (PAMPs), which are widely expressed by a variety of infectious microorganisms (Innate immunity), and the other category is damage‐associated molecular patterns (DAMPs).^139^ Toll‐like receptors (TLRs) expressed by enteroendocrine cells are one of the PRRs, and they are able to detect bacterial products and activate vagal afferents through a basal process called neuropod cell.^140^

In addition to inducing brain deposition of α‐synuclein through curli produced by intestinal bacteria, animals exposed to curli to produce bacteria had more TLR2, interleukin‐6, and tumor necrosis factor expression in the brain.^141^ Furthermore, curli has been confirmed to activate induced nitrogen monoxide synthase,^142^, ^143^ as well as nuclear factor‐kappa B (NF‐κB) signaling.^144^ Extracellular misfolded fibrillar α‐synuclein released from neural cells or oligodendrocytes is recognized by microglial TLR2 (as a heterodimer with TLR 1) as a PAMP or DAMP, which in turn activates downstream pathways involving myeloid differentiation primary response 88 and NF‐κB, further triggering the production of tumor necrosis factor and interleukin‐1β,^145^, ^146^ and finally damaging dopaminergic neurons.

Due to dysbacteriosis of the intestinal flora and/or bacterial overgrowth of the small intestine, as well as higher intestinal barrier permeability, over‐stimulation of the innate immune system occurred. The decreased abundance of *Prevotella* might lead to decreased mucin synthesis, which was associated with increased intestinal permeability, resulting in exposure to bacterial antigens and endotoxin and then inducing excessive α‐synuclein expression in the colon and even in the brain.^147^, ^148^ Disruption of the intestinal epithelial barrier could activate TLRs, which in turn activated downstream signaling pathways that promoted inflammation and oxidative stress in the gut and brain of PD patients.

Gut bacteria are capable of producing corresponding TLR ligands, such as LPS. As a pro‐inflammatory bacterial endotoxin derived from gut, LPS synthesis genes are verified to be higher in PD patients than those in normal controls.^149^ LPS acts as a TLR4 ligand and translocation of LPS could induce upregulation of local and systemic inflammatory responses, as well as excretion of pro‐inflammatory mediators, and thus cause severe neurodegeneration.^150^ Also, Enteric bacteria could produce other neurotoxic molecules (e.g., d‐lactate, ammonia) and neurotoxins (e.g., *Clostridium tetani*, *botulinum*, *butyricum*, *baratii*, etc.) and enter CNS via systemic or extrinsic afferent nerve fibers, leading to neuronal damage.^151^, ^152^

### 4.3 Endocrine system

Intestinal bacterial cells have the ability to generate a variety of neuroactive molecules, such as serotonin, catecholamines, glutamate, γ‐aminobutyric acid (GABA), and SCFAs.^153^, ^154^, ^155^ *Lactic acid bacteria* produced acetylcholine and GABA, while *E. coli* generated norepinephrine, 5‐hydroxytryptamine (5‐HT) and dopamine, and *streptococci* and *enterococci* produced 5‐HT. Activation of 5‐HT_4_ receptors induced maturation of ENS and modulated its adult function.^156^ Binding of serotonin to 5‐HT receptors on microglia induced the release of cytokine‐bearing exosomes involved in the secretion of certain cytokines.^157^ N‐Butyric acid produced by intestinal bacteria was involved in the regulation of energy homeostasis and stimulated adipocytes and induced the secretion of several neuropeptides, such as glucagon‐like peptide‐1. These neuropeptides could regulate insulin secretion, glucose and metabolism and food intake.^158^, ^159^

The HPA axis is a key neuroendocrine signaling system involved in physiological homeostasis and stress response, which regulates the physiological responses to stress, thereby driving the organisms to adapt their own behavior or environment to stress.^160^ Environmental stress and elevated systemic inflammatory cytokines stimulated the pituitary gland to secrete adrenocorticotropic hormone via corticotropin‐releasing factor from the hypothalamus, which in turn led to the release of cortisol from the adrenal gland. Cortisol is a major stress hormone that affects different human organs, such as the brain.^161^ Also, stress affected the growth of intestinal bacteria by enhancing the secretion of α‐defensin (an antimicrobial peptide) from Paneth cells.^162^ Meantime, acute stress induced a 3‐day‐delayed increase in colonic paracellular permeability which involved mast cell degranulation and overproduction of interferon‐γ. Norepinephrine stimulated the proliferation of a variety of enteric pathogens, such as non‐pathogenic *E. coli* isolates and pathogenic *E. coli* 0157: H7: 3, as well as overgrowth of *Pseudomonas aeruginosa* and increased virulence of *Campylobacter jejuni*.^163^, ^164^, ^165^, ^166^

**FIGURE 1:**
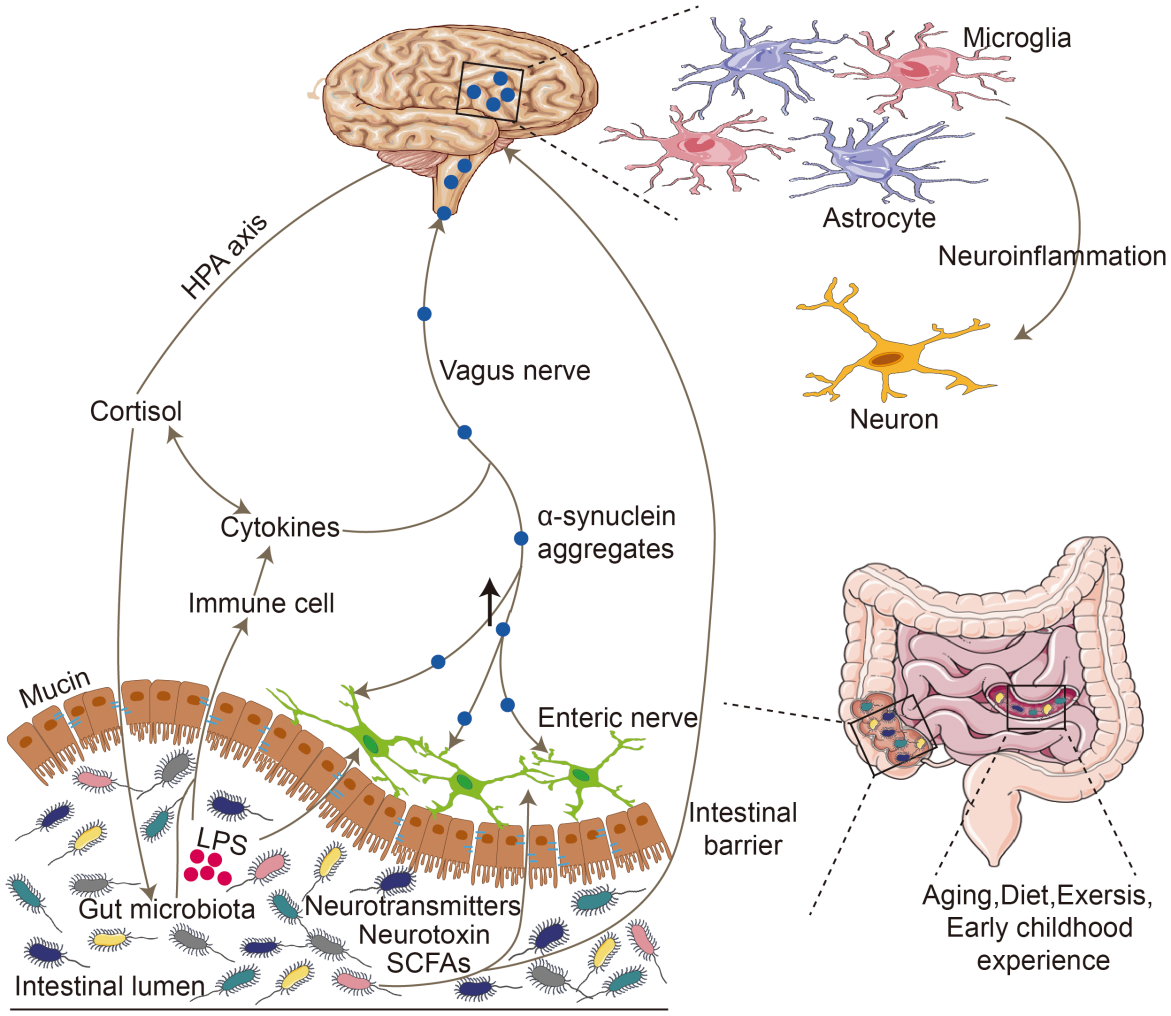
Gut microbiota were involved in PD pathogenesis through the gut‐brain axis. Within gut microbiota dysregulated, LPS and other neurotoxic molecules produced by the multiplication of pathogens penetrated the damaged gut barrier to contact immune cells and intestinal nerves. Cytokines secreted by immune cells then interacted with cortisol and participated in vagal communication between gut and brain. In addition, α‐synuclein thus produced by intestinal neurons was deposited in brain retrogradely through the vagus, activating glial cells and triggering neuroinflammation to damage dopaminergic neurons. At the same time, neurotoxic molecules could also penetrate the blood–brain barrier through the peripheral blood circulation into CNS to trigger dopaminergic neurodegeneration.

In general, the brain‐gut axis is mainly communicated through the cross information of the body's nervous, immune, and endocrine systems. As shown in Figure 1, gut microbiota is involved in the reciprocal communication of the gut‐brain axis and thus the pathogenesis of PD through this communication pathway.

## 5 INTERVENTION TREATMENT OF GUT MICROBIOTA

### 5.1 Fecal microbiota transplantation

Fecal transplantation (FMT) is a procedure in which a healthy donor's stool is placed into the gastrointestinal tract of another patient to restore normal bacterial composition by altering the host's dysbiosis. At present, FMT has been most commonly used to study the treatment of *Clostridium difficile*.^167^ In addition, FMT also appears to play a pivotal role in the interventional treatment of neurological disorders. For example, rapid improvement in AD symptoms was shown after FMT treatment for recurrent *Clostridium difficile* infection.^168^ In depression animal studies, FMT could improve depression‐like behavior and reduce intestinal inflammation and mucosal destruction in rats by regulating intestinal bacteria.^169^ Another recent clinical trial also indicated that after FMT treatment 11 PD patients presented an increase in *Blautia* and *Prevotella* and a dramatic decrease in Bacteroidetes abundance, improvement non‐motor symptom questionnaire, and constipation symptoms.^170^ Based on the above studies, the potential to develop FMT as an emerging treatment might be able to increase the selectivity of treatments for neurological diseases, including PD.

However, it should be noted that conclusions based on animal studies are limited in supporting FMT treatment. The conclusions based on clinical trials also lack credibility since the sample size was too small to satisfy the optimal study design. In addition, if treatment of FMT continued to be developed, we should be alert to the possible side effects of FMT, such as abdominal pain and distension.^171^ At the same time, after fecal transplantation is transformed into clinical practice, the volume and frequency of transplantation, microbial composition in feces, age of applicable patients, and type of applicable disease need to be further clarified.

### 5.2 Probiotic therapy

The International Scientific Association for Probiotics and Prebiotics (ISAPP) states that probiotics are defined as “live microorganisms which when administered in adequate amounts confer a health benefit on the host”.^172^, ^173^ In a clinical study, probiotics improved values of oxidative capacity as well as enhanced antioxidant responses in patients with IBD.^174^ Oral probiotics improved functional gastrointestinal disease.^175^ Most importantly, there were no significant side effects in the above clinical studies. Probiotics also played a beneficial role in PD‐associated diseases, such as diabetes and depression. Probiotics reduced the production of inflammatory factors and induced changes in microbial flora structure and increased the relative abundance of beneficial microorganisms in diabetic rats.^176^ In the treatment of depression, probiotics reduced chronic unpredictable mild stress‐induced depressive behavior of rats and increased the levels of norepinephrine and 5‐HT and inhibited the expression of adrenocorticotropic hormone and corticosterone.^177^ Consistent with this finding, in a clinical depression treatment trial, the symptoms of all clinical trial participants were improved with a significant correlation between *Ruminococcus gnavus* and depression.^178^ Thus, the current findings could consider probiotic development as an adjunct to existing treatments.

The association between probiotics and neurodegenerative disorders has been well studied in recent years. A large number of basic studies showed that probiotics were able to improve the symptoms of PD animal models. In detail, probiotics restored mitochondrial function and energy metabolism in rat PD model, thereby producing a protective effect on dopaminergic neurons.^179^, ^180^ Moreover, clinical studies demonstrated that probiotic supplementation in PD patients improved gene expressions of inflammatory factors and constipation.^181^, ^182^, ^183^, ^184^ In addition, probiotic administration for 16 weeks reduced dyskinesia in gait patterns, balance function, and motor coordination in mice.^185^ A randomized, double‐blind, placebo‐controlled clinical trial of 60 PD patients indicated that probiotics treatment for 12 weeks reduced UPDRS in PD patients.^186^ Thus, probiotics hold potential benefits with improving the motor and non‐motor symptoms of PD, and the neuroprotective effects of probiotics as potentially developed treatments warrant further exploration.

### 5.3 Antibiotic intervention

To date, studies have found that the pathogenic bacteria damaged the intestinal barrier and thus induced PD. For example, *H. pylori* infection and excessive growth of small intestinal bacteria impaired intestinal balance and had potential connections with neurodegenerative diseases. An antibiotic intervention is any compound that kills or inhibits the growth of microorganisms at low concentrations. The use of antibiotics to inhibit the multiplication of harmful bacteria is considered to be one of the effective adjunctive treatments for PD. In a meta‐analysis, eradication of *H. pylori* improved the symptoms of PD.^187^ Thus, eradication of *H. pylori* has been recommended for the potential combined therapeutic strategy during levodopa treatment of PD since it could improve drug bioavailability and reduce motor fluctuations.

An antibiotic mixture treatment for 14 days protected the striatum and signal‐to‐noise ratio from 1‐methyl‐4‐phenyl‐1, 2, 3, 6‐tetrahydropyridine‐induced mouse dopaminergic neurotoxicity, while this mixture reduced the diversity of host intestinal bacteria in mice and altered the composition of host intestinal microbiota at the genus and species levels.^188^ Similar results showed that treatment with broad‐spectrum antibiotics (neomycin, 2 mg/mL; vancomycin 0.2 mg) attenuated 6‐hydroxydopamine‐induced dopaminergic neuronal loss in SN of rats and motor dysfunction and reduced expression of pro‐inflammatory mediators in the striatum.^189^ At present, the neuroprotective effects of tetracycline,^190^ minocycline^191^ and rifampicin^192^ have been well demonstrated.

In clinical trials, correction of small intestinal bacterial overgrowth in PD patients with antibiotics improved gastrointestinal symptoms and motor fluctuations.^193^ Although current clinical studies suggest that exposure to certain types of oral antibiotics appeared to be associated with an increased risk of PD.^194^, ^195^ However, in the same year another study found no significant association between antibiotic use and the incidence of PD.^196^ Therefore, not only the conclusions from animal studies and human studies conflict, but also there are differences among the conclusions of human studies. The reasons for these controversial conclusions might be the subjects of case–control studies do not use antibiotics rationally to treat PD, and untargeted use and abuse of antibiotics play an active role in changing the composition of host intestinal bacteria and reducing the composition and diversity of host intestinal bacteria. Therefore, it is worth exploring more reasonable clinical studies in the future to clarify whether antibiotics generate neuroprotective potential.

## 6 CONCLUSION

So far, gut microbiota has been extensively studied for nearly two decades, but there are still new findings suggesting that the close association between gut microbiota and PD has not been fully illuminated. The future should not be limited to focusing only on the composition of gut microbiota, but rather on identifying important microbial genes. This could more accurately characterize bacteria from another perspective and is also more conducive to elucidating the involvement of gut microbes in PD pathogenesis, enhancing the potential of gut microbiota to predict PD and as a therapeutic target for PD.

## AUTHORS' CONTRIBUTIONS

All authors read, revised and approved the final manuscript.

## CONFLICT OF INTEREST

The authors declare that they have no competing interests.

## ETHICS APPROVAL AND CONSENT TO PARTICIPATE

Not applicable.

## CONSENT FOR PUBLICATION

Not applicable.

## Data Availability

Data sharing is not applicable to this article as no new data were created or analyzed in this study.
